# Fatal and non-fatal injuries due to intentional explosions in Nepal, 2008-2011: analysis of surveillance data

**DOI:** 10.1186/1752-1505-7-5

**Published:** 2013-03-20

**Authors:** Oleg O Bilukha, Kristin Becknell, Hugues Laurenge, Luhar Danee, Krishna P Subedi

**Affiliations:** 1 International Emergency and Refugee Health Branch, Center for Global Health, Centers for Disease Control and Prevention, 4770 Buford Hwy, MS F-60, Atlanta, GA, 30341, USA; 2 United Nations Children’s Fund, UN Building, Pulchowk, P.O. Box 1187, Kathmandu, Nepal

**Keywords:** Intentional explosions, Improvised explosive device, Injury, Nepal, Armed violence

## Abstract

**Background:**

Nepal is one of the post-conflict countries affected by violence from explosive devices. We undertook this study to assess the magnitude of injuries due to intentional explosions in Nepal during 2008-2011 and to describe time trends and epidemiologic patterns for these events.

**Methods:**

We analyzed surveillance data on fatal and non-fatal injuries due to intentional explosions in Nepal that occurred between 1 January 2008 and 31 December 2011. The case definition included casualties injured or killed by explosive devices knowingly activated by an individual or a group of individuals with the intent to harm, hurt or terrorize. Data were collected through media-based and active community-based surveillance.

**Results:**

Analysis included 437 casualties injured or killed in 131 intentional explosion incidents. A decrease in the number of incidents and casualties between January 2008 and June 2009 was followed by a pronounced increase between July 2010 and June 2011. Eighty-four (19.2%) casualties were among females and 40 (9.2%) were among children under 18 years of age. Fifty-nine (45.3%) incidents involved one casualty, 47 (35.9%) involved 2 to 4 casualties, and 6 involved more than 10 casualties. The overall case-fatality ratio was 7.8%. The highest numbers of incidents occurred in streets or at crossroads, in victims’ homes, and in shops or markets. Incidents on buses and near stadiums claimed the highest numbers of casualties per incident. Socket, sutali, and pressure cooker bombs caused the highest numbers of incidents.

**Conclusions:**

Intentional explosion incidents still pose a threat to the civilian population of Nepal. Most incidents are caused by small homemade explosive devices and occur in public places, and males aged 20 to 39 account for a plurality of casualties. Stakeholders addressing the explosive device problem in Nepal should continue to use surveillance data to plan interventions.

## Background

Explosive devices continue to maim, kill, and terrorize civilian populations worldwide despite new and continuing efforts to prevent injury and assist victims, increasing attention from various global actors, and novel prohibitions and limitations in formal and customary humanitarian law [1]. During 2011 alone, Action on Armed Violence’s Explosive Violence Monitoring Project (EVMP) recorded 30,127 casualties of explosive devices from 2,522 incidents in 68 countries worldwide [2]. Of particular concern is the disproportionate civilian burden of fatal and non-fatal injuries: 71% casualties recorded by EVMP in 2011 were civilians. EVMP reported that such a preponderance of injuries among civilians is common “across a range of explosive weapons types, delivery methods, and intended targets” [2].

In recent years, explosive violence has received increasing attention on the global stage. In 2009, for example, United Nations (UN) Secretary-General Ban Ki-moon stated that he was “increasingly concerned at the humanitarian impact of explosive weapons, in particular when used in densely populated areas” and encouraged UN member states to take measures to address this issue [3]. The following year, the Secretary-General urged all relevant parties to support “more systematic data collection and analysis of the human costs of [explosive device] use” in order to better understand “the humanitarian impact of these weapons” [4].

Nepal is one among many post-conflict countries affected by violence from explosive devices. The 1996-2006 armed conflict between the Government of Nepal and the Communist Party of Nepal (Maoist) (CPN/M) resulted in an estimated 12,000 deaths and the displacement of over 100,000 persons [5]. Although the Comprehensive Peace Agreement (CPA) was signed in November 2006 and the CPN/M has since entered the parliamentary process and intermittently joined the government, political tensions remain high. Dozens of armed groups have emerged following the signing of the CPA and are active in certain regions of the country, especially in the southern region of Nepal (Terai belt) [6]. Persistent post-conflict explosive violence perpetrated with the intent to kill, maim, and terrorize is attributed to multiple factors, including political instability, proliferation of politically and criminally motivated armed groups, increased access to explosive devices, discrimination based on ethnicity and religion, poverty, unemployment, and poor security [7-9].

Survivors of explosive violence endure physical pain, critical psychological sequelae, and disability. Families of casualties face significant socioeconomic losses, and affected communities suffer from the destruction of critical infrastructure, environmental consequences, and collective psychological and social trauma [8,10]. In Nepal as a whole, explosive violence poses a threat to the peace and reconciliation process and impedes economic and social development [8,10]. Unfortunately, there are no published peer reviewed studies describing the epidemiology of intentional explosion incidents and casualties in Nepal, and therefore very limited data are available to guide interventions to address this problem.

We undertook this study to assess the magnitude of fatal and non-fatal injuries due to intentional^a^ explosions in Nepal during 2008-2011 and to describe time trends and epidemiologic patterns for these events.

## Methods

Data on injuries due to intentional explosions in Nepal were obtained from the United Nations Children’s Fund (UNICEF) and the Informal Sector Service Center (INSEC). INSEC is a Nepali non-governmental organization (NGO) with the primary mission of promoting policies, institutions and capacity that contribute to the protection and promotion of human rights. INSEC personnel collect injury surveillance data and manage the casualty database. The database included injuries that occurred over a four-year period between 1 January 2008 and 31 December 2011.

INSEC implemented nationwide prospective surveillance for injuries due to intentional explosions in January 2008 in collaboration with UNICEF. INSEC supported staff members called “District Representatives” (DRs) in each of Nepal’s 75 districts. DRs produced “Human Rights (HR) reports” on a wide range of human rights violations (e.g., incidents of kidnapping, intentional explosions, torture, extrajudicial killing, etc.) occurring in their districts. INSEC surveillance staff monitored HR reports mentioned above as well as local and national media in Nepali and English for intentional explosion incidents that resulted in human casualties and abstracted data on victim demographics and incident dates, locations and circumstances from these reports. Active community-based surveillance began in October 2009, when injury data collection form was introduced and DRs were trained in casualty data collection using this form. DRs monitored community networks (e.g., local police, district administrators, village-level authorities, local media and partner NGOs) to identify incidents of intentional explosions as soon as they occurred. After obtaining each initial incident report, the respective DR visited the site and interviewed the victim, family member of the victim, or eyewitness using a standardized data collection form mentioned above. Information collected included the incident date and location, victim demographics, incident circumstances (e.g., type of setting where the incident occurred, type of explosive device that caused the incident), and whether the injury resulted in death. Pictures of different explosive devices were used to help the victim identify the type of device causing the incident. Verbal informed consent was obtained prior to each interview.

**Figure 1:**
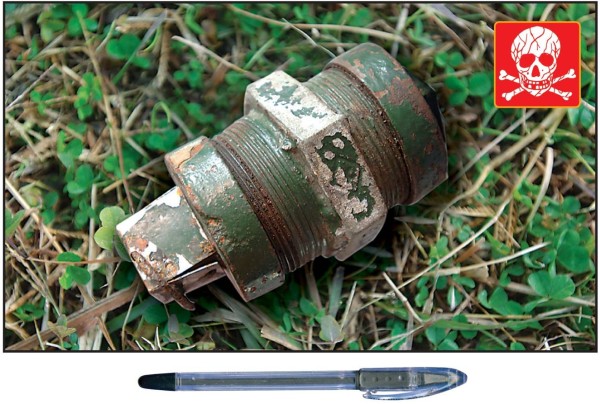
Socket bomb.

**Figure 2:**
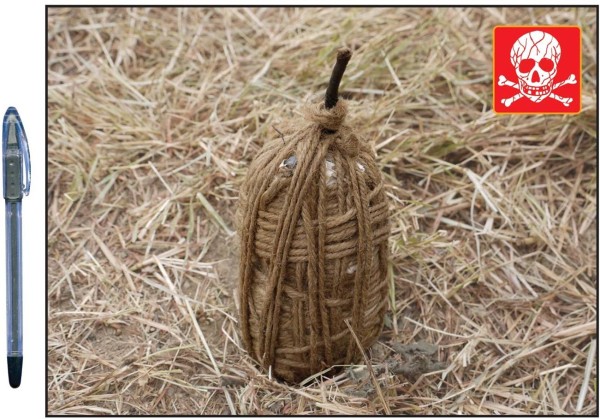
Sutali bomb.

**Figure 3:**
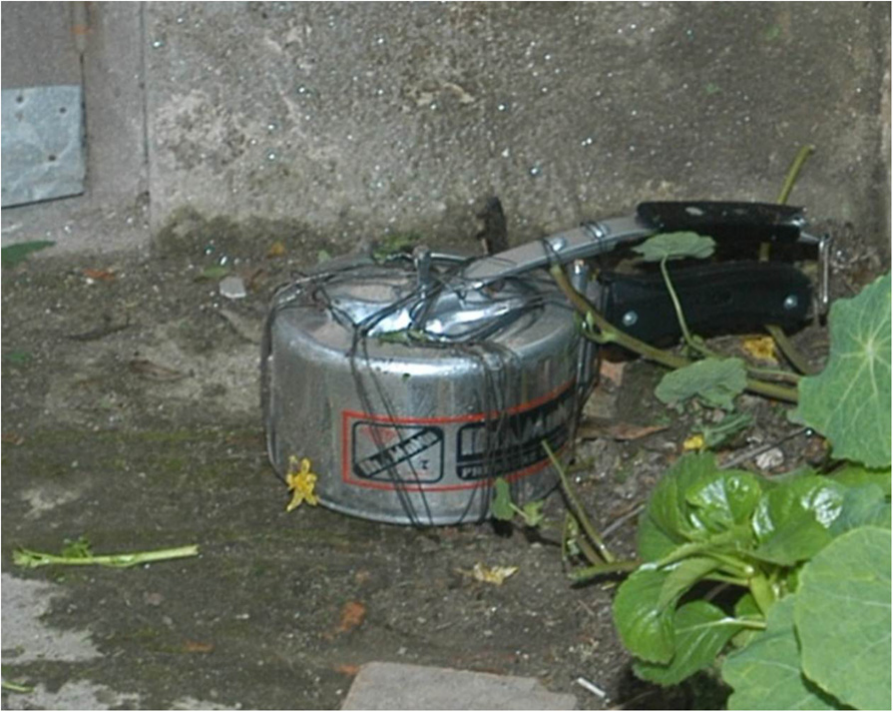
Pressure cooker bomb.

The case definition used by the surveillance system included casualties injured or killed by explosive devices knowingly activated by an individual or a group of individuals with the intent to harm, hurt or terrorize. The case definition excluded casualties of unintentionally activated explosive devices (for example, injury from stepping on a landmine or injury from tampering with abandoned ordnance); victims injured directly during active fighting (e.g., those injured by bullets, artillery or rocket projectiles, or aerial bombing); casualties injured by explosive devices used for fishing, hunting, road construction or other purposes where the intent was not to harm or terrorize; and casualties with minor injuries that did not require medical treatment. “Incident” was defined as the explosion of an improvised explosive device (IED) or other explosive device resulting in one or more casualties as defined above. “IED” was defined as a homemade (as opposed to industrially manufactured) explosive device. Examples of IEDs commonly used in Nepal include socket bombs (improvised hand grenades made of galvanized pipe sockets, Figure 1), sutali bombs (small explosive devices wrapped in rope, Figure 2), and pressure cooker bombs (pressure cookers filled with explosives, Figure 3).

A trained data manager checked the database for duplicate entries by comparing victim demographics and incident times and locations. Statistical analyses were performed using JMP software (release 9.0.1, SAS Institute Inc., Cary, NC). The Institutional Review Board of the Centers for Disease Control and Prevention exempted this study from review because it involved secondary analysis of routinely collected surveillance data used for programmatic purposes. Personal identifiers were not included in the final data set used for analysis.

## Results

**Figure 4:**
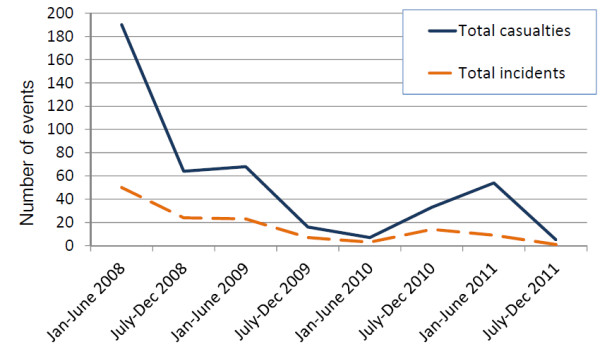
Time trends in numbers of casualties and incidents caused by intentional explosions in Nepal, 2008-2011.

**Figure 5:**
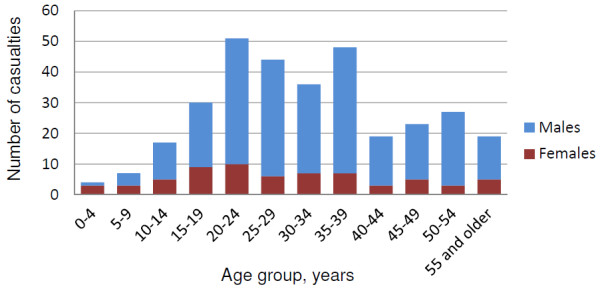
Age and sex distributions of fatal and non-fatal injuries caused by intentional explosions in Nepal, 2008-2011.

Analysis included 437 casualties injured or killed in 131 intentional explosion incidents between January 2008 and December 2011. Fifty-nine (45.3%) incidents involved one casualty, 47 (35.9%) involved 2 to 4 casualties, 19 (14.5%) involved 5 to 10 casualties, and 6 involved more than 10 (11, 13, 14, 14, 26 and 41) casualties (Table 1). Time trends in the numbers of casualties and incidents are presented in Figure 4. Following a decrease in the number of incidents and casualties between January 2008 and June 2009, there was a pronounced increase between July 2010 and June 2011. Overall, 341 (78.0%) injuries were among males and 84 (19.2%) were among females; the sex of 12 (2.7%) casualties was unknown. 286 (65.4%) casualties were adults, 40 (9.2%) were children (under 18 years of age), and the age of 111 casualties (25.4%) was unknown (Table 2). The mean age of casualties with known age was 31.6 years (standard deviation 13.5; range: 2 to 70 years). The distribution of injuries by age group and sex is presented in Figure 5. The highest number of injuries occurred among individuals between 20 and 39 years of age. The overall case-fatality ratio was 7.8%.

**Table 1:** Distribution of incidents due to intentional explosions by surveillance type, Nepal, 2008-2011 (N = 131)

	**Media surveillance**	**Active surveillance**	**Total incidents**
	**Jan 08 –Sep 09**	**Oct 09 – Dec 11**	**N (% total)**
	**N (% total)**	**N (% total)**	
**Type of explosive device**			
Socket bomb	22 (22.0)	14 (45.2)	36 (27.4)
Sutali bomb	23 (23.0)	4 (12.9)	27 (20.6)
Pressure cooker bomb	3 (3.0)	2 (6.5)	5 (3.8)
Other improvised explosive device	11 (11.0)	6 (19.4)	17 (13.0)
Unknown	41 (41.0)	5 (16.1)	46 (35.1)
**Place of incident**			
Busy street or crossroads	25 (25.0)	6 (19.4)	31 (23.7)
Shop or market	10 (10.0)	5 (16.1)	15 (11.5)
Place of worship	5 (5.0)	0 (0)	5 (3.8)
Near government or police office	5 (5.0)	1 (3.2)	6 (4.6)
Near school or hospital	4 (4.0)	0 (0)	4 (3.1)
Near stadium	2 (2.0)	0 (0)	2 (1.5)
Factory or industry	6 (6.0)	1 (3.2)	7 (5.3)
On the bus	2 (2.0)	5 (16.1)	7 (5.3)
Near bus station	3 (3.0)	0 (0)	3 (2.3)
Home	17 (17.0)	8 (25.8)	25 (19.1)
Other	20 (20.0)	5 (16.1)	25 (19.1)
Unknown	1 (1.0)	0 (0)	1 (0.8)
**Number of persons injured or killed**			
One	44 (44.0)	15 (48.4)	59 (45.3)
Two	19 (19.0)	5 (16.1)	24 (18.3)
Three to four	18 (18.0)	5 (16.1)	23 (17.6)
Five to ten	16 (16.0)	3 (9.7)	19 (14.5)
More than ten	3 (3.0)	3 (9.7)	6 (4.6)
**Total**	**100**	**31**	**131**

**Table 2:** Distribution of casualties due to intentional explosions by surveillance type, Nepal, 2008-2011 (N = 437)

	**Media surveillance**	**Active surveillance**	**Total casualties**
	**Jan 08 –Sep 09**	**Oct 09 – Dec 11**	**N (% total)**
	**N (% total)**	**N (% total)**	
**Sex**			
Male	255 (78.0)	86 (78.2)	341 (78.0)
Female	60 (18.3)	24 (21.8)	84 (19.2)
Unknown	12 (3.7)	0 (0)	12 (2.7)
**Age group**			
Child (< 18 years)	25 (7.6)	15 (13.6)	40 (9.2)
Adult	192 (58.7)	94 (85.5)	286 (65.4)
Unknown	110 (33.6)	1 (0.9)	111 (25.4)
**Outcome**			
Non-fatal injury	298 (91.1)	105 (95.5)	403 (92.2)
Fatal injury	29 (8.9)	5 (4.5)	34 (7.8)
**Type of explosive device**			
Socket bomb	50 (15.3)	48 (43.6)	98 (22.4)
Sutali bomb	47 (14.4)	7 (6.4)	54 (12.4)
Pressure cooker bomb	16 (4.9)	6 (5.5)	22 (5.0)
Other improvised explosive device	87 (26.6)	41 (37.3)	128 (29.3)
Unknown	127 (38.8)	8 (7.3)	135 (30.9)
**Place of incident**			
Busy street or crossroads	69 (21.1)	15 (13.6)	84 (19.2)
Shop or market	26 (8.0)	18 (16.4)	44 (10.1)
Place of worship	26 (8.0)	0 (0)	26 (5.9)
Near government or police office	28 (8.6)	1 (0.9)	29 (6.6)
Near school or hospital	5 (1.5)	0 (0)	5 (1.1)
Near stadium	44 (13.5)	0 (0)	44 (10.1)
Factory or industry	7 (2.1)	2 (1.8)	9 (2.1)
On the bus	9 (2.8)	52 (47.3)	61 (14.0)
Near bus station	18 (5.5)	0 (0)	18 (4.1)
Home	43 (13.1)	16 (14.5)	59 (13.5)
Other	50 (15.3)	6 (5.5)	56 (12.8)
Unknown	2 (0.6)	0 (0)	2 (0.5)
**Total**	**327**	**110**	**437**

Intentional explosion incidents occurred in 27 of 75 districts in Nepal. Overall, 85.5% of incidents (112 of 131) and 86.7% of injuries (379 of 437) occurred in the southern region of Nepal (Terai belt). Of nineteen incidents that occurred outside of Terai belt, nine took place in Kathmandu and its vicinity (Kathmandu and Lalitpur districts), accounting for 59.9% of all injuries (33 of 58) that occurred outside of Terai belt.

The highest number of incidents occurred in streets or at crossroads (31 or 23.7%), in victims’ homes (25 or 19.1%), and in shops or markets (15 or 11.5%). Other common places of incidents included industrial settings (7), buses (7), government and police offices (6), places of worship (5), schools and hospitals (4), and stadiums (2) (Table 1). Incidents on buses and near stadiums claimed the highest numbers of casualties per incident (61 casualties in 7 incidents and 44 casualties in 2 incidents, respectively).

Socket, sutali, and pressure cooker bombs caused the highest numbers of incidents (36 [27.4%], 27 [20.6%], and 5 [3.8%], respectively, Table 1); other types of IEDs caused 17 (13.0%) incidents. The type of explosive device was unknown in 46 (35.1%) incidents (Table 1).

Almost all the records with missing data on sex, age, place of incident and type of explosive device (except one casualty with missing age and five incidents with missing explosive type) were collected prior to October of 2009, when surveillance data were collected solely from media sources (Tables 1, 2).

## Discussion

Numbers of intentional explosive incidents and casualties in Nepal have decreased considerably since early 2008. However, the overall situation in Nepal remains fragile, as illustrated by the pronounced increase in both incidents and casualties between July 2010 and June 2011.

Most incidents in this study occurred in public places—streets and crossroads, shops and markets, industrial settings, buses, and places of work and worship. Intentional explosive incidents in public places have been common in other settings as well. 85% of deaths resulting from 68 intentional explosions in Istanbul between 1976 and 2000, for example, were due to incidents in public places [11]. Of 138 children injured in intentional explosions in Israel during a 15-month period in 2000-2001, 54% were injured in incidents on the road and 36% were injured in incidents in public or commercial buildings [12].

Almost one fifth of incidents in this study took place in the home, a finding which is also corroborated by data from other settings. Incidents in homes accounted for 15.0% of 120 intentional explosion-related deaths in Istanbul [11]. An earlier study of victim-activated explosive events in Nepal between 2006 and 2010 found that almost 40%—nearly double the proportion in the current study—took place in the home [13].

Over 70% of incidents in our study for which the devices are known were caused by sutali and socket bombs, which are relatively small homemade explosives. These same types of explosive devices were used in over 60% of victim-activated incidents occurring in Nepal between July 2006 and June 2010 [13].

Overall, incidents resulting in fewer casualties were more common than incidents resulting in more casualties: 45.3% of incidents involved only one casualty, while only 4.6% resulted in 10 or more casualties. This is similar to findings for 138 victim-activated explosive incidents in Nepal, of which 54.3% of incidents involved only one casualty and only 1.4% resulted in 10 or more casualties [13]. This trend is common to some other settings as well. Of 77 explosive events in Northern Ireland, for example, only 17% resulted in 6 or more casualties and only 3 incidents resulted in more than 20 casualties [14]. On the other hand, data from other countries indicate higher numbers of casualties per incident; in a study of 32 explosive events in Israel, for example, 25% of the incidents resulted in more than 25 casualties [15].

Our dataset revealed an average of 3.3 casualties per incident, which is relatively low compared to average numbers in other settings. A review of 14 published studies of 220 intentional explosive incidents worldwide showed an average of 15.3 casualties per incident [16], whereas individual country studies in Northern Ireland, Indonesia, France, Turkey, and Israel reported average values of 4.4 [14], 7.3 [17], 24.4 [18], 51.8 [19], and 63.3 [20], respectively.

The overall case-fatality ratio in the current dataset is 7.8%, which is lower than that of an earlier analysis of victim-activated injuries in Nepal (13.7%) [13] and analyses in several other countries—some of which have better developed health services than Nepal. Analyses of multiple explosive incidents found average case fatality ratios of 12.4, 14.3, 16.1, 31.3, and 39.7% in Turkey [19], Colombia [21], Israel [22], Thailand [23], and Indonesia [17], respectively.

Data from this study and studies in other settings indicate that numbers of casualties per incident and case-fatality ratios are affected by explosive device type [24]. The predominant use in Nepal of small improvised devices such as socket and sutali bombs, with relatively small explosive charges, likely influences the relatively low numbers of casualties per incident and case-fatality ratio. Likewise, casualties per incident in Israel decreased threefold between 1975 and 1979, assumed by Adler et al. to result from smaller explosive charges used due to tighter security measures [25].

The physical locations of incidents are also likely to affect numbers of casualties per incident and case-fatality ratios. In the current study, incidents on buses and near stadiums claimed the highest numbers of casualties per incident (61 casualties in 7 incidents and 44 casualties in 2 incidents, respectively). Multiple studies have shown that incidents in buses result in the most lethal types of injuries and the highest mortality rates [15,20,22,26], while many others have discussed how the physical location of an explosion can affect the type, anatomical location, and severity of injuries [16,24,26]—which, in turn, affect numbers of casualties per incident and case-fatality ratios.

Almost 4 out of 5 casualties in this study were men, a proportion higher than that found in an earlier study of victim-activated explosive injuries in Nepal (69.4% male overall, 61.6% male among adults) [13] but similar to the sex distribution in studies of intentional explosions in several other settings. Seventy-one percent of 339 casualties injured in intentional explosions in Northern Ireland between 1972 and 1980 were male [14], whereas 81.7% of 120 people fatally injured in intentional explosions in Istanbul between 1976 and 2000 were male [11]. These data suggest that males in these places had a much higher risk of injury than females, likely because explosive devices were deployed and detonated in settings and at times where and when males were more likely to be present than females.

Of injuries in this study for which age is known, over 55% occurred in individuals aged 20-39 years and 9.2% were among children (under 18 years of age). This finding contrasts with data from victim-activated explosions in Nepal, in which 55% of injuries occurred among children and the highest numbers of injuries occurred among individuals 5 to 19 years of age [13]. However, the findings of the current study are similar to those in other studies of intentional explosions. A study of 511 casualties at one Israeli hospital between 1975 and 1979 showed that over 40% of injuries occurred among people aged 19-40 [25], and an analysis of national data in Israel between 2000 and 2003 found that 49.2% and 21.1% of casualties were 15-29 and 30-44 years old, respectively [15]. Analysis of 120 explosive event deaths in Istanbul between 1976 and 2000 found that 50% of those injured were 21-30 years of age [11]. The mean age of 31.6 years for the current study is also similar to means found in other settings: 30.9, 34.5, and 34.8 years in Jerusalem [27], Paris [18], and Istanbul [11], respectively. Over 45% of all casualties in our study for whom age and sex are known were males aged 20-39, indicating that males in this age group are at the highest risk of injury.

Surveillance of intentional explosive device injuries varies widely among countries. In some countries, hospital and ambulance records are sufficiently complete to conduct complex analyses, as in Israel [28], Northern Ireland [14], and Thailand [23]. Systems such as the Israel National Trauma Registry [12,15] and the Arson and Explosives National Repository [29] and Bomb Data Center [30] in the United States serve as national repositories for intentional explosion data. The RAND Corporation, the Oklahoma City National Memorial Institute for the Prevention of Terrorism [31], and the United States Department of State [32] collect information on explosive incidents worldwide, although their data likely are undermined by the poor capacity of many countries to collect information about such incidents.

National governments, health care facilities, and other stakeholders with fewer resources may have difficulty collecting data on intentional explosive incidents. A special report on Indonesia described the difficulty of collecting precise explosive injury data because hospitals do not maintain disaster plans and do not always record patient data properly [17]. Describing two explosive incidents in Karachi, Umer et al. remind us that the quality of care in some developing countries varies with social status, meaning that injury victims with lower social status may not receive care in the best—if any—hospitals [33]. Such considerations must be taken into account when considering venue-based surveillance data. Another barrier to reporting in all countries, regardless of resources, lies in the difficulty of defining intentional explosive incidents as an injury category [34] and the lack of standardized definitions [35]. Variations in explosive incident characteristics such as device type, activation circumstances, and context (including armed conflict, criminal activity, and intent to cause terror among civilian populations) often lead to inconsistencies in definitions and reporting practices.

Given its ability to overcome many of the challenges faced by lower-income countries and its inclusion of data points that are omitted from many data collection systems, Nepal’s active community-based surveillance system may serve as a model for other resource-challenged settings. To our knowledge, this is the only functioning active community-based surveillance system for intentional explosion injuries. Because it collects data from many sources we can assume that it possesses a relatively high degree of sensitivity. Furthermore, prospective data collection soon after incidents is likely to lower the possibility of recall bias. The availability of victim-activated explosive injury data from an overlapping time period in Nepal [13], collected in the exact same manner, afforded us the ability to compare and contrast trends from victim-activated and intentional explosions in a similar setting.

This study is subject to several limitations. Early use of media reporting for the surveillance system may mean that data are missing or incorrect (due to use of potentially biased or inaccurate media reports) between January 2008 and the start of active surveillance in 2009. The system may also underestimate the magnitude of intentional explosive injury due to the likely non-detection of some cases and the exclusion of combatants from data collection.^b^ Furthermore, the use of self-reported data is subject to reporting bias.

## Conclusions

Despite the overall decrease of injuries resulting from intentional explosions during the study period, intentional explosion incidents still pose a threat to the civilian population of Nepal. Most incidents are caused by small homemade explosive devices and occur in public places, and males aged 20 to 39 account for a plurality of casualties. Nepali government bodies, UNICEF, and other partners addressing the explosive device problem should continue to use surveillance data to plan interventions. Additional study of similarities and differences between intentional and victim-activated explosive devices could lead to improvement in prevention and response activities. Further examination of how factors such as injury type and anatomical location, setting, and the age and sex of casualties predict injury outcomes—and comparison with non-explosive traumatic injuries—could help medical professionals better understand how to treat individuals injured in intentional explosion events.

### Ethics approval

Exempted from review by the Institutional Review Board of the Centers for Disease Control and Prevention as the primary intent of surveillance was determined to be non-research. The study constitutes a secondary analysis of surveillance data routinely collected for programmatic purposes.

## Endnotes

^a^Some injury specialists classify any injury caused by an explosive device—regardless of whether the device is activated by the victim or another party—as intentional because such devices are manufactured and deployed with the intent to cause physical harm or terror. In this article we use the term “intentional” to describe explosive events and injuries knowingly activated by individuals and groups in order to maintain consistency with definitions used by the surveillance system in Nepal and to differentiate between these events and those caused by victim-activated explosions.

^b^The burden of injuries caused by intentionally deployed explosive devices is also likely underestimated because some IEDs that are intentionally deployed or launched fail to explode as intended. Unless cleared by security forces, such devices left in public places remain a threat to civilians and may cause victim-activated explosion injuries if detonated by tampering or other victim activities.

## Abbreviations

CPA: Comprehensive peace agreement; CPN/M: Communist party of Nepal (Maoist); DR: District representative; EVMP: Explosive violence monitoring project; IED: Improvised explosive device; INSEC: Informal sector service center; NGO: Non-governmental organization; UN: United Nations; UNICEF: United Nations children’s fund

## Competing interests

The authors declare that they have no competing interests, financial or otherwise.

## Authors’ contributions

OOB and HL designed the study, HL, LD and KPS collected the data, OOB analyzed the data, OOB and KB interpreted the data, OOB and KB drafted the manuscript, HL, LD, and KPS critically revised the manuscript for important intellectual content. OOB is a guarantor. All authors read and approved the final manuscript.

## Disclaimer

The findings and conclusions in this report are those of the authors and do not necessarily represent the views of the Centers for Disease Control and Prevention and the United Nations Children’s Fund.
